# A Multilevel Physically Based Model of Recrystallization: Analysis of the Influence of Subgrain Coalescence at Grain Boundaries on the Formation of Recrystallization Nuclei in Metals

**DOI:** 10.3390/ma16072810

**Published:** 2023-03-31

**Authors:** Peter Trusov, Nikita Kondratev, Matvej Baldin, Dmitry Bezverkhy

**Affiliations:** 1 Department of Mathematical Modeling of Systems and Processes, Perm National Research Polytechnic University, 614990 Perm, Russia; 2 Laboratory of Multilevel Structural and Functional Materials Modeling, Perm National Research Polytechnic University, 614990 Perm, Russia

**Keywords:** multilevel modeling, thermomechanical processing, dynamic recrystallization, subgrain coalescence, material structure, subgrain structure, grain boundaries

## Abstract

This paper considers the influence of subgrain coalescence at initial high-angle boundaries on the initiation and growth of recrystallization nuclei (subgrains) under thermomechanical treatment. With certain processing regimes, adjacent subgrains in polycrystalline materials can be assembled into clusters during coalescence. Subgrain clusters at high-angle boundaries are the preferred potential nuclei of recrystallization. Coalescence is one of the dynamic recovery mechanisms, a competing process to recrystallization. When intensive coalescence develops on both sides of the grain boundary, recrystallization slows down or even stops. The problem formulated is solved using a multilevel modeling apparatus with internal variables. Application of the statistical multilevel model modified to take into account the local interaction between crystallites makes it possible to explicitly describe dynamic recrystallization and recovery. The results of modeling the behavior of a copper sample are presented and the effects of temperature, deformation velocity and subgrain structure on the formation and growth of recrystallization nuclei at arbitrary and special grain boundaries during coalescence are analyzed.

## 1. Introduction

The thermomechanical processing of polycrystalline materials has found wide industrial application. The metal and alloy forming processes are often multistage, for instance severe plastic deformation alternates with subsequent heat treatment [1,2]. The application of complex material structure formation modes makes it possible to provide the necessary macro properties of a final product. Thus, the microstructure control of the materials undergoing this treatment is a key and relevant problem for materials science and engineering [3,4]. Many thermally activated processes, among which the most significant are recrystallization, solid-state phase transitions and recovery [5,6,7], occur due to different thermal and thermomechanical effects. These processes bring about changes in the structure of materials subjected to thermomechanical treatment, and thus an adequate control of these changes enables designing materials with the unique effective characteristics of polycrystalline materials [4,8,9,10].

In context of the grain structure evolution, the most significant changes appear as a result of recrystallization [11,12,13,14,15]. Primary recrystallization leads to the formation of low-defect grains in the deformed material and the subsequent migration of high-angle boundaries (or their segments) driven by the energy stored on the defects during inelastic deformation [16,17,18,19]. During the process of recrystallization, the shape, dimensions and orientation of the crystallographic coordinate system of new grains changes with respect to the deformed material [7,11,18]. This type of structure is called a recrystallized structure.

It can be concluded that a theoretical and experimental study of recrystallization with a consideration of changes in the subgrain structure is of special practical interest. The phenomenon of recrystallization is multi-scale in nature; it occurs at several levels of polycrystalline material implementation. Since the experimental research methods are expensive, this generates a need to use mathematical modeling methods for studying this problem [16,20,21]. Physically based mathematical modeling that relies on a multilevel approach with internal variables is an effective tool for solving this complicated problem [22,23]. Multilevel physical models involving the explicit consideration of the material structure evolution are used extensively to describe dynamic recrystallization [24,25,26,27]. Certain variables and parameters were introduced in these models to describe the material structure and the mechanisms governing the formation of recrystallization nuclei and the migration of grain boundaries [19,25,28,29]. In the context of a physical approach, three main classes of multilevel models [22] are widely met: statistical [27,30], self-consistent [26,31] and direct [32,33]. Direct models explicitly consider the topology of grains and, during numerical implementation they are oriented towards the use of a finite element method. Although these models are more accurate compared to the others, they are very resource-intensive, which gives no way of studying the manufacture of real structures in technological processes. At present, models based on the self-consistent approach [26,31] have gained broad recognition. Self-consistent models (the grain environment is replaced in these models by the effective medium) are less computationally expensive compared to direct models, but they cannot be used study the local interactions between adjacent structural elements [34]. In this connection, a compromising solution is to use advanced statistical models because they are computationally fast and permit analyzing contact interactions between crystallites.

The purpose of this work is to study the influence of coalescence on the formation of recrystallization nuclei. Coalescence-related rotations occur due to the local interactions of a subgrain with adjacent subgrains. Therefore, the formulated problem is solved in the framework of the advanced statistical model of inelastic deformation, which permits the consideration of the interactions between adjacent structural elements (subgrains, grains) [27,34]. The modeling of coalescence for a representative volume of subgrains with consideration of their geometry as similar to real three-dimensional geometry is a laborious and complex task [35,36,37]. Most models of recrystallization that deal with subgrain coalescence are simplified models. They cannot provide an explicit description of the contact local interactions between subgrains [28,38] and/or are applicable to one- or two-dimensional crystalline materials [3,35,39,40].

Previously, we have developed a method to consider subgrain coalescence by means of an advanced statistical model of inelastic deformation [34]. As the main driving force of coalescence, a reduction in the internal energy of the considered representative volume of subgrains, including the energy of subgrain boundaries, was taken [41]. Coalescence was modeled explicitly via use of a polyhedral subgrain structure. In the statistical model proposed by the authors [34], the high-angle boundary was not considered and its influence on the coalescence process was, accordingly, not taken into account. In this study, a high-angle grain boundary that affects the coalescence of subgrains and the formation of recrystallization nuclei is introduced explicitly into consideration. The purpose of this study is to consider the influence of coalescence on the formation and growth of recrystallization nuclei in the framework of the extended statistical model of inelastic deformation, designed to explore materials with both arbitrary high-angle and special (with reduced energy) grain boundaries.

## 2. Mechanisms of Subgrain Structure Evolution under Thermomechanical Effects

Recrystallization in metals and alloys is a well-known phenomenon; reviews of theoretical and experimental studies of this process can be found in a great variety of papers [11,12,13,14,16,42]. Grain boundary engineering (GBE) techniques are used to design materials with an increased proportion of special boundaries; GBE is based on the recrystallization or is accompanied by it [10,43]. Special boundaries attract the attention of researchers because the polycrystals with a large number of such boundaries show improved corrosion resistance, high fracture strength and long-life fatigue properties [44,45].

One of the central problems in the study of recrystallization is to provide a deeper insight into the physics of the formation and growth of recrystallization nuclei [18,46,47,48]. Nucleation is generally observed in the crystal regions with orientation gradients (grain or twin boundaries, transition bands, junctions of deformation bands) [14,18,46,49,50]. Recrystallization is accompanied by the fine subgrain structure evolution caused by the recovery action [6,51,52,53,54], which manifests itself most vividly in materials with high stacking fault energy (SFE). Upon recovery, the density of crystal defects decreases, and subgrain structures with reduced energy are formed [7,13]. Depending on the type and mechanism of recrystallization, different elements of these structures (usually cells and subgrains) are frequently associated with nuclei [42,46,49,51,53,55,56]. A recrystallization nucleus is understood as a small, low-defect, region of a crystal capable of stable growth in at least one direction in the deformed material [46,49]. The formation of recrystallization nuclei has been studied for a relatively long time [18,42,49,51,55], but currently there is no unified theory that addresses various aspects of this process and corresponding physical models [12,13,46]. This is because of a great number of different factors, including the characteristics of materials (impurity atoms, SFE, particles of secondary phases, deformation modes, texture, initial grain size, etc.) and the impact parameters (temperature, deformation velocity) related to the formation and growth of recrystallization nuclei [7,15,16,52,57]. For this reason, the mechanisms governing the nucleation of recrystallized grains have been extensively discussed in the literature and there are different classifications of these mechanisms [7,18,49,57]. Variation in the factors listed above can change these mechanisms and, in some cases, the type of recrystallization [14,15,58,59]. Most of the mechanisms of nucleation of recrystallized grains described in the literature are similar to each other and represent different variants of the formation of subgrains or groups of subgrains which tend to become nuclei. These considerations lead to the idea of finding universal mechanisms responsible for the formation of recrystallization nuclei.

Despite extensive theoretical [13,38,46] and experimental [14,57,60] studies and much progress in understanding the recrystallization and accompanying processes gained in the last decade, some mechanisms of material nucleation under arbitrary thermomechanical loading still remain unclear. In this paper, the mechanism of recrystallization driven by the migration of pre-existing high-angle boundary segments during plastic deformation and by the strain induced boundary migration (SIBM) is investigated [15,17,46,49,50,52,60,61,62,63]. Many researchers have reported that the formation of recrystallization nuclei is closely related to the subgrain structure evolution. For instance, subgrain coarsening at grain boundaries results in the formation of nuclei and in the implementation of discontinuous recrystallization following the SIBM mechanism [7,18,46,52]. One possible subgrain coarsening mechanism is coalescence (considered in this paper) and another is low-angle boundary migration [7,49,52,53,60,64]. During the coalescence process, adjacent subgrains merge together due to the gradual disappearance of a common boundary of subgrains experiencing rotation [41,65,66]. Coalescence can take place not only at grain boundaries, but also inside grains. The main characteristic of materials for coalescence is SFE. At large deformation, the subgrain merging inside grains provokes the formation of coarse subgrains, the misorientation of which with the surrounding material increases, and thus they become recrystallization nuclei [7,12,13,24,49]. This type of continuous recrystallization is not considered here.

The thermomechanical processing of polycrystalline materials gives rise to the evolution of a subgrain structure, which affects the recrystallization process. Cahn was among the first to suggest that the low-angle boundaries occur due to the excess density of lattice dislocations of the same sign (polygonization process) [55]. According to his ideas, the subgrain structure elements formed in such a way as to act as recrystallization nuclei. Later, the theory of subgrain structure evolution for materials with high and medium stacking fault energy was further developed by Kuhlmann-Wilsdorf and colleagues [67,68,69]. The background to the outlined theory is that the low-energy structures formed during plastic deformation occur due to a decrease in the energy stored in defects in the material. In the framework of the Kuhlmann-Wilsdorf theory, three scale levels were distinguished [69]: (1) the cells separated by incidental dislocation boundaries or dislocation cell boundaries; (2) the cell blocks distinguished by dense dislocation boundaries or microbands; (3) domains separated by corresponding domain boundaries. The listed subgrain structure boundaries are dislocation and low-angle boundaries. The generated 3D cell structure consists of tangles [7,54]. The recovery process includes the annihilation of excess dislocations and the rearrangement of the remaining ones into the low-energy structures, which are the regular dislocation networks or the low-angle boundaries of tangled cell walls [7,46,54]. Concurrently, the cells transform into subgrains; the characteristic size of the cell and subgrain are approximately the same and are equal to 1 μm.

**Figure 1:**
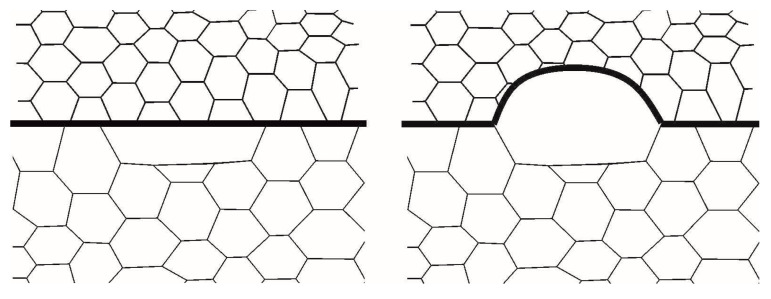
Scheme of the formation of a recrystallization nucleus according to the SIBM mechanism (the drawing is based on the scheme given in [46]).

In this study, the mechanism of recrystallization nuclei formation is considered. This mechanism is based on the migration of grain boundary regions initially pre-existing in a polycrystal due to strain-induced boundary migration (SIBM). Beck and Sperry were probably the first to experimentally investigate this mechanism by testing high purity polycrystalline aluminum samples under conditions of preliminary rolling and subsequent annealing [61]. Further studies confirmed that this mechanism can be encountered in a great variety of polycrystalline materials (aluminum, copper, magnesium, silver, nickel alloys, steels, etc.) [17,45,52,60,63,70,71,72,73]. The implementation of SIBM is schematically shown in Figure 1. It can be seen that, after the completion of pre-existing plastic deformation and the fulfillment of energy criterion, a certain grain boundary segment bulges. After this segment migrates, the material with low defect density remains behind it. Part of the material adjacent to the migrating boundary segment is usually associated with a large subgrain [13,17,46]. This subgrain is a recrystallization nucleus, and it can be separated from the parent grain during subsequent thermomechanical treatment [52,74]. Since the subgrain has a slight misorientation relative to the parent grain, SIBM is reliably detected in the experiments by electron backscatter diffraction (EBSD), orientation imaging microscopy (OIM) and transmission electron microscopy (TEM) [7,15,46,60]. Under this recrystallization mechanism, the texture of the recrystallized material slightly differs from that of the deformed material [7,46,74]. The SIBM mechanism realized in the vicinity of initial grain boundaries is typical of relatively small deformations of order 20% [7,17,74]. The continued inelastic deformation causes recrystallization nuclei to occur preferentially at such grain substructures as transition bands [11,18,46], twin boundaries [46,75,76], and deformation band boundaries [46,74].

The kinetic description of the SIBM mechanism obtained from the analysis of existing experimental results was formulated for the first time by J.E. Bailey [17,62]. It was established in [17,62] that a boundary segment begins to bow into the adjacent, more defective, grain provided that a decrease in the local volume energy associated with defects elimination is greater than or equal in absolute value to an increase in the grain boundary energy due to the growth of the boundary area during the formation of recrystallized grains [17,62]:

(1) $$f^{\left(i,j\right)}=e_{dst}^{\left(i,j\right)}-e_{gb}^{\left(i,j\right)}\Delta s/\Delta v\geq 0$$

where Δs is the growth of the boundary area when the subgrain (recrystallization nucleus) volume changes by Δv, $e_{dst}^{\left(i,j\right)}$ is the difference in specific stored energies per unit volume between the adjacent subgrains *i* and *j*, and $e_{gb}^{\left(i,j\right)}$ is the specific grain boundary energy per unit area between the *i*-th and *j*-th subgrains. Criterion (1) states that large subgrains have the growth advantage.

**Figure 2:**
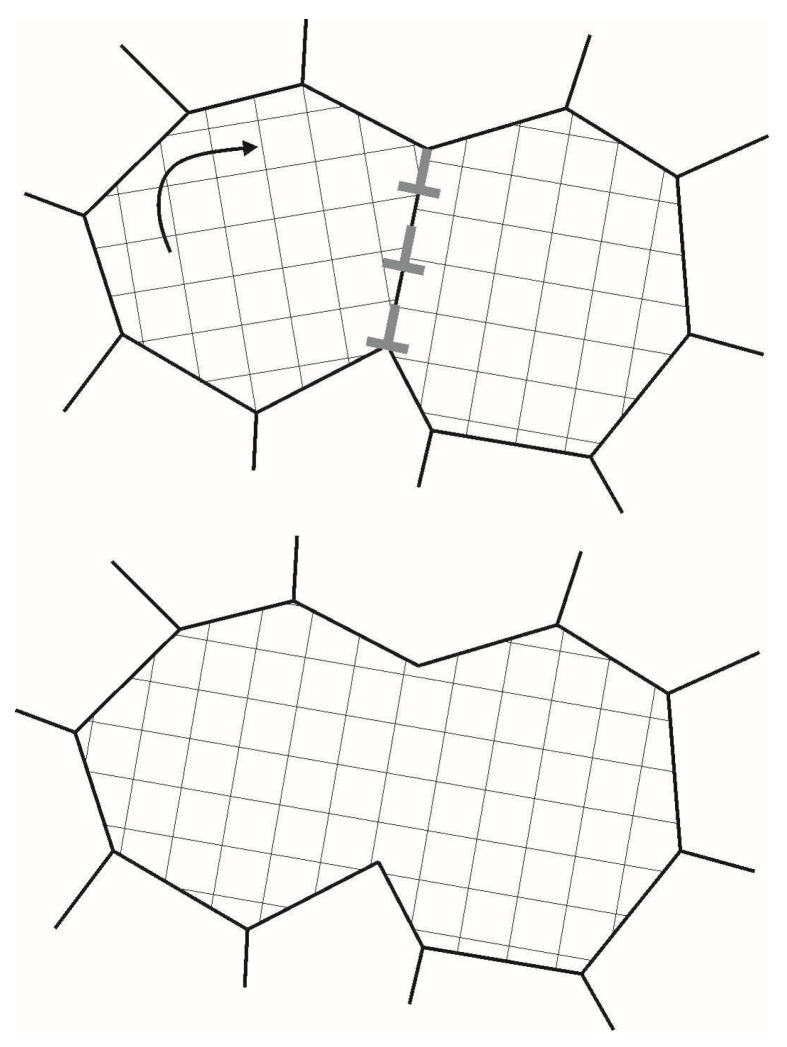
Scheme of subgrain rotation at coalescence (based on the scheme from [41]).

Subgrain growth occurs under two mechanisms: (1) sub-boundary migration, and (2) subgrain coalescence [7,49,52,53,60,64]. We study here how coalescence affects the coarsening of subgrains at grain boundaries. The process of coalescence is associated with the dissociation of the low-angle boundary of adjacent subgrains. At coalescence, the adjacent subgrains acquire the same orientation; they can be considered as a single subgrain, and thus we can speak of the merging of subgrains (Figure 2). The driving force behind this process is surface energy reduction. The thermodynamic and kinetic aspects of subgrain coalescence were formulated in [41]. It can be seen that the surface energy of a subgrain experiencing rotation should decrease during coalescence [41]:

(2) $$E_{sb}=\sum_{i=1}^{N_{f}}s^{\left(i\right)}e_{sb}^{\left(i\right)}$$

where $s^{\left(i\right)}$ is the area of the subgrain boundary *i*-th facet, $e_{sb}^{\left(i\right)}$ is the specific (per unit area) surface energy of the subrain boundary *i*-th facet, and $N_{f}$ is the number of facets of the considered subgrain. The further description of the coalescence phenomenon is extended to include an analysis of the physical mechanisms of dissociation of a small-angle boundary [7,64,66,77,78]. The coalescence mechanism associated with the climb of dislocations and their annihilation in the subgrain boundary is likely to appear at small-angle boundaries with weak misorientation [7,77,78]. The multilevel model modified to take coalescence into account was described in [34].

Theoretical and experimental studies confirm that coalescence contributes much to the coarsening of subgrains at high-angle boundaries and to their subsequent rearrangement into recrystallized grains [15,41,52]. Doherty and Cahn were the first who discovered that coalescence occurs at high-angle boundaries [18]. Later, similar rotations were found experimentally [15,52,64]. Therefore, there is a need for physical modeling of subgrain coalescence and its influence on the formation of recrystallization nuclei. Subgrain coalescence that occurs by the elimination of a common low-angle boundary, the misorientation of which with an adjacent grain (separated by the high-angle boundary) increases, is an energetically favorable process [41]. This can be seen by taking the Reed–Shockley relation as a basis for describing the surface energy of the low-angle boundary [79]:

(3) $$e_{sb}\left(\varphi \right)=\{\begin{array}{ll} e_{sb0}\varphi \left(a-\ln\varphi \right), & \varphi \leq \varphi_{m},\\ e_{sb0}\varphi_{m}, & \varphi >\varphi_{m}, \end{array}$$

where the following notation is used: φ is the angle of mutual misorientation between adjacent subgrains, $\varphi_{m}$ is the mutual misorientation angle corresponding to the maximum value of $e_{sb}$, $e_{sb0}$, and a denotes the Reed–Shockley parameters determined experimentally. It was assumed in (3) that the value of energy for the arbitrary high-angle boundary is independent of the misorientation angle [7,73,80]. In accordance with (3), $de_{sb}/d\varphi$ is minimum at high-angle boundaries, which explains the energetic advantage of coalescence in these sites. Thus, coalescence promotes the generation of large subgrains, which, under criterion (1), are energetically profitable for the formation of recrystallization nuclei.

**Figure 3:**
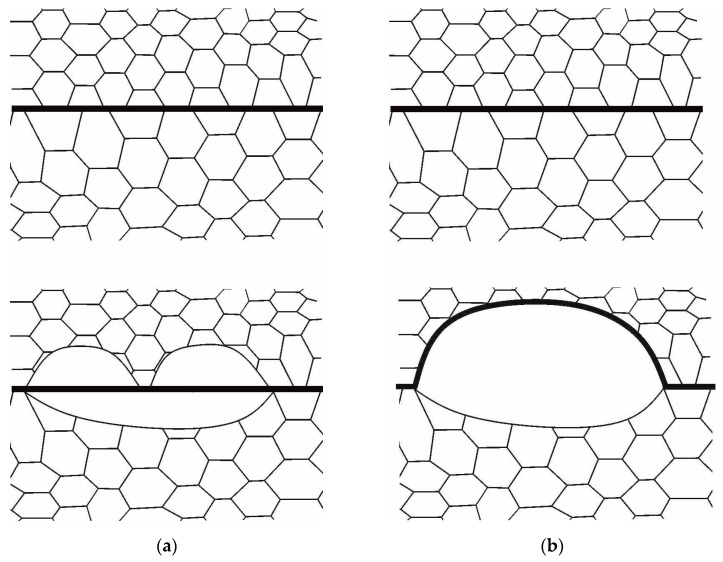
Scheme of the formation of a recrystallization nuclei at high-angle boundaries [52]: (**a**) intensive coalescence in adjacent grains and (**b**) in one of the grains.

The physical mechanism associated with the effect of coalescence on the formation of recrystallization nuclei at high-angle boundaries and the microstructural studies confirming its existence were considered in [52]. It is believed that the main reason for the formation of recrystallization nuclei is the difference in stored energy on the initial grain boundary segments following the SIBM mechanism. It was stated in [52] that lattice dislocations form networks that produce a subgrain structure inside grains. Dislocation networks at high-angle boundaries are accommodated by these boundaries, which causes their annihilation. The interaction between the network dislocations and the grain boundary intensifies the coalescence process. Therefore, it is reasonable to expect that subgrain coalescence will occur at crystallite parts near the high-angle boundaries (Figure 3). Figure 3a shows how subgrain coalescence develops in both adjacent grains. Since coalescence causes the stored energy and defect density to decrease in two adjacent grain boundary segments, the local recrystallization criterion (1) is not fulfilled and a new recrystallized grain is not formed. The scenario from Figure 3a is an example of competition for stored energy between the dynamic recovery and recrystallization processes. Figure 3b illustrates the case of intense coalescence for one of the grains represented schematically in the bottom part of the figure. At this event, the large subgrain formed due to coalescence has the energy advantage of forming a recrystallization nucleus. Then, the size of this subgrain increases during two processes: (1) subgrain coalescence in the parent grain, and (2) migration of the high-angle boundary into the adjacent grain. The rate of high-angle boundary migration is much higher than the rate of coalescence-driven subgrain growth [28,52]; thus, the penetration of the recrystallized grain deep into the defective grain proceeds more actively (Figure 3b).

## 3. Materials and Methods

**Figure 4:**
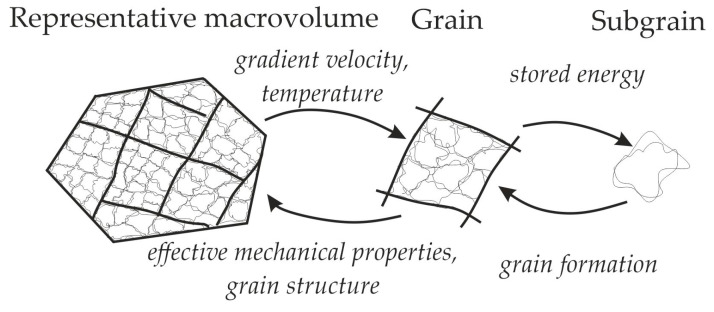
Scheme: scale levels and relation between the multilevel model structural elements (thin lines—subgrain boundaries, thick lines—grain boundaries) [27].

The problem formulated in our study was solved using the multilevel statistical model of inelastic deformation modified to consider the local interaction between structural elements (grains, subgrains). A detailed description of the model with regard to dynamic recrystallization is provided in [27,30]. In the model, three structural-scale levels are distinguished: macrolevel, mesolevel-I, and mesolevel-II (Figure 4). The macrolevel refers to a representative volume of the polycrystal containing a statistically significant number of grains (mesolevel-I elements); a grain consists of homogeneous subgrains (mesolevel-II elements). At mesolevel-I, the problem of determining the stress–strain state of a grain is solved in the framework of the extended statistical model, and the corresponding internal variables of the model are obtained. The closed mathematical formulation of the mesolevel-I model is given in [27]. At the macrolevel, the effective properties of the material are determined, and the macrolevel effects are transferred to the underlying scale levels. Mesolevel II (a single subgrain) is an auxiliary level that is used to adequately analyze the recrystallization SIBM mechanism and the subgrain coalescence when modeling the formation of nuclei at grain boundaries.

In the volume of one grain, subgrains are weakly misoriented relative to each other, and hence the energy stored on the defective structure $e_{st}$ is assumed to be approximately the same in all subgrains of a single grain. The difference in stored energy for the subgrains belonging to different grains and having a common high-angle boundary is significant. The data on the stored energy $e_{st}^{I}$, which were calculated at mesolevel-I using the multilevel model, are transferred to mesolevel-II [27]:

(4) $$e_{st}^{II}\approx e_{st}^{I}\overset{def}{=}e_{st}$$

where $e_{st}^{II}$ is the energy stored at mesolevel-II. The fulfillment of criterion (1) is verified at mesolevel-II.

The modification of the multilevel statistical model so that it can be applied to consider coalescence was described in [34]. To take into account the local interactions of adjacent subgrains, a polyhedral subgrain structure was formed in the freely distributed Neper software [81]. The geometry of this structure was determined by the following model variables: the subgrain volume $v_{sb}$, the characteristic size $d_{sb}$ (given by the diameter of a sphere of equivalent volume $v_{sb}$), and the parameters of the flat boundary segments (facets)—normal $n_{sb}^{\left(j\right)}$ and area. The link between the adjacent subgrains and the considered subgrain was shown. These data were transferred to the calculation module of the statistical model. The method similar to that used here for the formation of a grain structure was presented in [82]. To describe the plastic deformation-driven subgrain rotation with increasing dislocation density, the model of the crystallographic coordinate system (CCS) rotation was applied. This model is based on the evolution of the incidental low-angle boundaries due to the “trapping” of an excess density of dislocations of the same sign on these boundaries. A description of this model can be found in [34,83,84].

It is assumed that coalescence (Figure 2) between two adjacent subgrains occurs when the energy criterion is fulfilled and time limit is to be set [34]. According to this energy criterion, the total subgrain surface energy $E_{sb}$ should reduce because of the rotation $r_{m}$, which combines the CCS of the considered subgrain with that of the adjacent subgrain, i.e., the inequality [34]:

(5) $$E_{sb}^{r_{m}}\left(t\right)\leq E_{sb}\left(t\right)$$

must be fulfilled. Here, $E_{sb}^{r_{m}}$ is the subgrain surface energy after the imposed rotation $r_{m}$. The coalescence process is a diffusion process that develops due to the subgrain boundary dissociation caused by the rearrangement of dislocations in the sub-boundary and by the growth of a distance between them. Therefore, it is necessary to generate coalescence at the finite critical time $t_{c}$ (started at the beginning of the treatment process), which should be long enough to complete the process, but it should not exceed the current time t. The value of $t_{c}$ is determined by the relation [34]:

(6) $$t_{c}=\frac{s}{21e_{sb0}B_{p}b}\ln(\ln\left(\frac{\varphi_{m}}{\varphi_{0}}\right)/\ln\left(\frac{\varphi_{m}}{\varphi_{c}}\right)),$$

where s is the area of the considered subgrain, $e_{sb0}$ and $\varphi_{m}$ are the Reed–Shockley parameters, $\varphi_{0}$ is the minimally possible angle in (6) (the calculated value of the angle is 0.000069 radians [78]), b is the Burgers vector modulus in the material, $B_{p}=\frac{2b^{3}}{skT}D_{0}\exp\left(-\frac{Q_{p}}{RT}\right)$ is the dislocation climb mobility with regard to pipe diffusion, k is the Boltzmann constant, T is the absolute temperature, R is the universal gas constant, and $Q_{p}$ is the activation energy of pipe diffusion. For $t_{c}$, the estimation expression (6) was obtained via integrating the subgrain rotation rate dφ/dt during coalescence at cooperative dislocation climbing [41] with consideration of pipe diffusion and inhomogeneous boundary dislocation distribution [78].

**Figure 5:**
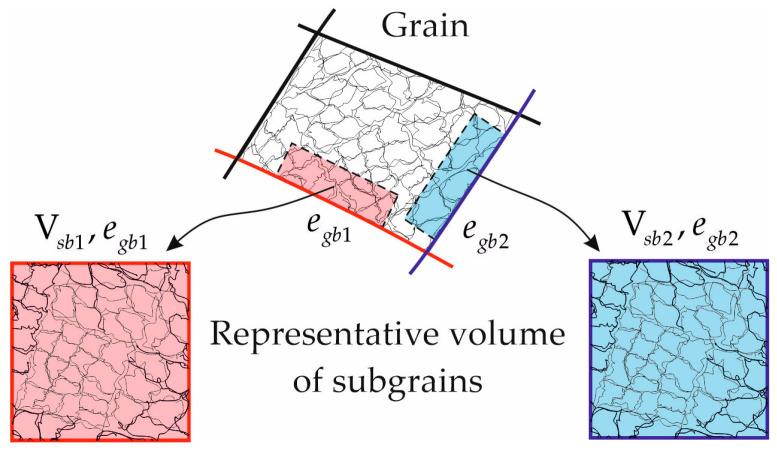
Scheme of the formation of a representative volume of subgrains for the grain boundary facet under consideration.

To model coalescence at the high-angle boundary facet $e_{gb}$, a representative volume of subgrains is considered and a polyhedral structure, the external shape of which corresponds to the cube of volume $V_{sb}$, is constructed in Neper. It is assumed that all cubes’ faces are associated with the considered high-angle grain boundary and have the same grain energy $e_{gb}$ (Figure 5).

In the representative volume of subgrains, coalescence causes a decrease in the dislocation density (Figure 3) and, accordingly, a release of stored energy. Thus, coalescence and recrystallization are the “competing” processes in terms of the energy stored in defects. If coalescence arises on both sides of the grain boundary, then the conditions for recrystallization following the SIBM mechanism may not be satisfied (Figure 3a). To take this effect into account, the energy released due to coalescence should be determined, and the recrystallization criterion defined by (1) should be modified. A distinguishing feature of the model is that the stored energy is determined at mesolevel-I, and thus it is averaged over the volume of the considered grain (4). For the same volume of subgrains V_*sb*_ at the grain facet, the energy *E*_*cl*_, decreasing locally during coalescence, is calculated as:

(7) $$E_{cl}\overset{def}{=}\int_{S_{sb}^{r_{m}}}e_{cl}dS_{sb}^{r_{m}},$$

where $e_{cl}$ is the specific surface energy released due to coalescence in the considered volume $V_{sb}$. The quantity $e_{cl}$ coincides with the specific surface energy of dissociated low-angle boundaries. In relation (7), integration is undertaken over the actual configuration of subgrains at the end of the coalescence process. In this case, the specific value of stored energy ${\hat{e}}_{st}$ (referring to the considered volume $V_{sb}$) at grain facet is determined with consideration of coalescence by the relation:

(8) $${\hat{e}}_{st}=\frac{\int_{V_{sb}}e_{st}dV_{sb}-\int_{S_{sb}^{r_{m}}}e_{cl}dS_{sb}^{r_{m}}}{\int_{V_{sb}}dV_{sb}}$$

Relation (8) takes into account that the volume of the material does not change during coalescence. The Bailey–Hirsch criterion (1) is applied to a recrystallization nucleus, i.e., an individual subgrain. If this criterion is fulfilled, then the subgrain is assumed to be a new recrystallized grain. Thus, the first term in (1) is understood as the quantity ${\hat{e}}_{dst}^{\left(i,j\right)}$, namely, the difference in specific stored energies per unit volume between the adjacent subgrains *I* and *j* with regard to the coalescence-induced energy release. The quantities Δs, Δv also change during coalescence. By Δs, Δv, we mean the quantities which correspond to the clusters of merging subgrains (Figure 3). In the developed model, the dynamic recovery effect is taken into account not only in the coalescence-induced energy release, but also, implicitly, in the amount of stored energy determined by hardening intensity. It is also assumed that the material in the reference configuration is annealed, and hence the initial value of stored energy in the considered representative volume of subgrains $V_{sb}$ is determined, according to the Reed–Shockley relation (3), by the energy of subgrain boundary defects.

**Figure 6:**
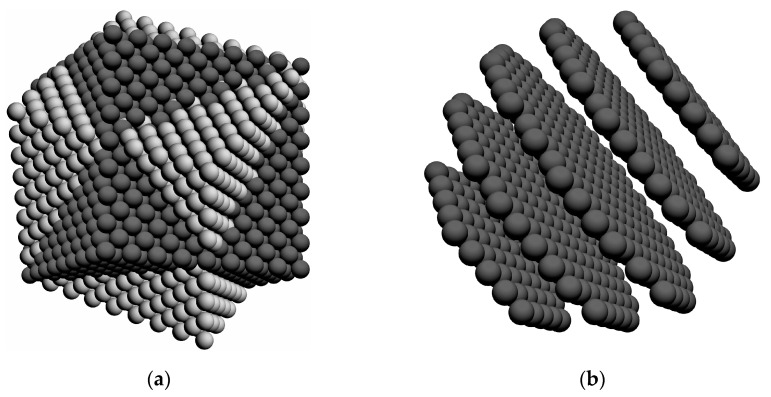
(**a**) Face-centered crystal lattices misoriented towards the [111] direction by the 60° angle; (**b**) superlattice which corresponds to this special orientation.

Under certain modes of thermomechanical processing, the proportion of special boundaries in polycrystalline materials increases [10,43,44,45]. That is the reason why our investigation addresses both the influence of high-angle boundaries on the formation of recrystallization nuclei and the special high-angle boundaries with reduced grain energy. Although the effect of mutual orientation on grain boundary mobility is a relevant issue [7,38], it is beyond the scope of this paper. In our early work, a method for determining special grain boundaries was developed in the framework of the statistical model of inelastic deformation, and the coincident-site lattice (CSL) model was modified [85]. The misorientation between two lattices around a common crystallographic axis by a certain angle results in the coincidence of some sites, which then form their own “superlattice”—a coincident site lattice [86] (Figure 6).

The main characteristic of CSL is the density of coincident sites $\Sigma^{-1}$, which is defined as the ratio of matching sites to all lattice sites. The modification of the CSL model described in [85] is based on the fact that one lattice is misoriented (by a fixed angle) with respect to another about a specific common crystallographic direction. It was shown in [87] that the densities of coincidence sites $\Sigma^{-1}$ are approximately the same for the boundary layer (the atoms of both lattices are in this layer), and for the volume of atoms of both lattices (the boundary layer is not identified). Thus, in the considered volume, the density of coincident sites $\Sigma^{-1}$ of a given orientation is calculated to determine the energy of the grain boundary. The relative value of grain boundary energy $e_{gb}^{\prime }$ is calculated from the following relation [85]:

(9) $$e_{gb}^{\prime }=1-\Sigma^{-1}$$

High-angle boundary migration occurs at the elevated deformation temperatures, which ensures its mobility. The mobility m depends on temperature following an Arrhenius-type law [7]:

(10) $$m=m_{0}\exp\left(-\frac{Q_{m}}{RT}\right)$$

where $Q_{m}$ is the activation energy of the grain boundary migration, and $m_{0}$ is the pre-exponential obtained experimentally. The high-angle boundary migration rate $v_{m}$ is determined by the product of the driving force f (the indices of neighboring crystallites are omitted) and the mobility m [7]:

(11) $$v_{m}=fm$$

Upon the fulfillment of criterion (1), the recrystallization nuclei become active and can be identified as new grains, which are assumed to be low-defective. Thus, all internal variables of the recrystallized grain correspond to the reference configuration in the state of the annealed material, except for the new grain orientation determined by the tensor o and the grain shape geometry. It is assumed here that the shape of nuclei penetrating into recrystallized grains is spherical and that every new recrystallized grain completely penetrates into the adjacent grain. In this case, $v_{m}$ is equal to the rate of change of the sphere radius r describing the recrystallized grain shape, i.e., $\dot{r}=v_{m}$ (the dot above the quantity indicates the material derivative). The absorbed grain volume is reduced by the volume of recrystallized grains. The recrystallized material volume fraction $X_{r}$ in the polycrystal under study is determined by the following relation:

(12) $$X_{r}=\frac{V_{r}}{V_{0}},V_{r}=\sum_{i=1}^{N_{r}}v_{r}^{\left(i\right)}$$

where V_0_ is the initial representative volume of the polycrystal, $V_{r}$ is the recrystallized material volume, $N_{r}$ is the number of recrystallized grains, and $v_{r}^{\left(i\right)}=\frac{4}{3}\pi r^{\left(i\right)}{}^{3}$ is the volume of the *i*-th sphere of the recrystallized grain. Note that the initial volume of the recrystallized grain coincides with that of the recrystallization nucleus (subgrain) for which criterion (1) has been fulfilled.

## 4. Results and Discussion

In this study, the inelastic deformation on the example copper bicrystal was investigated. Parameters for describing recrystallization were determined in [27], and for coalescence and rotation of subgrains in [34]. Hardening law parameters and initial critical stresses were found in [34] via analyzing the results of uniaxial compression tests on polycrystalline copper: (1) at temperature 300 K and deformation velocity 10^−3^ s^−1^ [88], and (2) at temperature 300 K and deformation velocity 2·10^−3^ s^−1^ [89]. For the high-temperature experiment [89], the hardening model parameters were determined prior to the active stage of dynamic recrystallization, i.e., at about 20% deformation. Computational experiments performed at deformation values obtained before the onset of active dynamic recrystallization, i.e., at 10–15% deformation, were also considered.

The developed statistical two-level mathematical model was based on the Voigt hypothesis, where the kinematic effects are specified by the velocity gradient $\hat{\nabla }V$ at the macrolevel. Temperature effects are transferred from the macrolevel, meaning the temperature T is a given temperature. The quasi-uniaxial deformation defined by the following law was investigated using the law

(13) $$\hat{\nabla }V=\dot{\varepsilon }k_{01}k_{01}-\frac{\dot{\varepsilon }}{2}k_{02}k_{02}-\frac{\dot{\varepsilon }}{2}k_{03}k_{03}$$

where $k_{0i}=k_{0}^{i}$ is the orthonormal basis of the laboratory coordinate system and $\dot{\varepsilon }$ is the prescribed deformation velocity. An analysis of the results given in [34] indicates that the intensity of coalescence depends significantly on the deformation velocity $\dot{\varepsilon }$ and temperature T.

The initial subgrain polyhedral structure was constructed in the Neper software package. To do this, the statistical distribution laws for subgrain sizes $d_{sb}$ and sphericity $\psi_{sb}$ were obtained. It was assumed that, in the reference configuration, the subgrain sizes were distributed according to the Rayleigh law, with a 0.25 μm mean value [29]. For the subgrain sphericity $\psi_{sb}$, a hypothesis for the uniform distribution with a high mean value of $\langle \psi_{sb}\rangle$ = 0.90 in the interval from 0.85 to 0.95 was accepted [34]. As noted above, consideration was given to a representative subgrain volume corresponding to the layer near the high-angle boundary facet. The data on the sizes of subgrains undergoing coalescence were transferred to the recrystallization submodel to verify whether the criterion was fulfilled and to determine the volume fraction of a recrystallized material.

**Figure 7:**
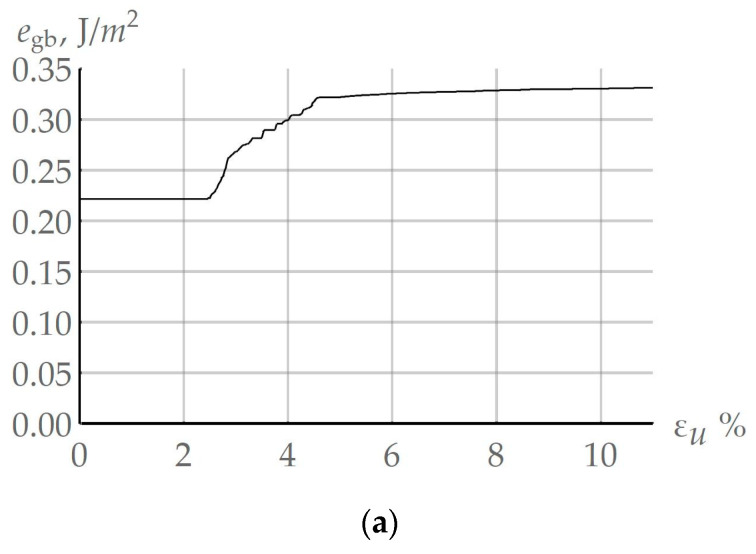

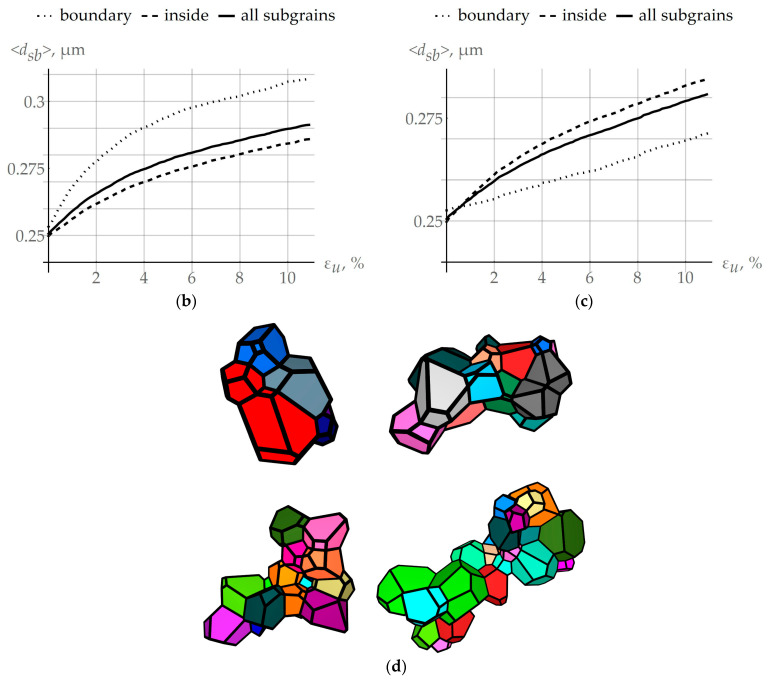
(**a**) Evolution of the grain boundary energy, which corresponds, in the reference configuration, to the special boundary at 60° [111]; (**b**) evolution of the linear mean size of subgrains $<d_{sb}>$ placed in different grain parts with respect to the high-angle boundary for high-angle and (**c**) special boundaries; (**d**) evolution of the subgrain cluster during coalescence at high-angle boundary, obtained in the numerical quasi-uniaxial deformation experiment ($\dot{\varepsilon }$ = 10^−3^ s^−1^, T = 550 K).

For high-angle boundaries, the sensitivity of surface energy to small changes in the misorientation angle was weak, and hence it can be neglected. Pursuant to the developed model, coalescence is realized more intensively at grain boundaries. To confirm this phenomenon and to compare the coalescence events at high-angle and special boundaries, a computational quasi-uniaxial deformation experiment with a copper bicrystal (deformation velocity $\dot{\varepsilon }$ = 10^−3^ s^−1^ and temperature T = 550 K) was carried out. In the reference configuration, the special boundary $\Sigma_{3}$ corresponds to the mutual misorientation of the grains rotated by an angle of 60° with respect to the general direction [111]. According to (9), the value of the energy of this grain boundary was 0.225 J/m^2^. As the grains deform, they undergo rotations determined by the applied rotation model [83], and the initial special boundary becomes the high-angle grain boundary with a small number of coinciding sites. Figure 7a illustrates the evolution of the grain boundary energy for the initial special boundary 60° [111]; at 5% deformation, the special boundary ceases to be such and its energy increases to that of the high-angle boundary. Figure 7b,c presents the dependencies of the linear mean size of subgrains <*d_sb_*> of the considered grain with high-angle (Figure 7b) and special $\Sigma_{3}$ (Figure 7c) boundaries. In accordance with the identified parameters [34], the high-angle grain boundary energy $e_{gb}$ was 0.337 J/m^2^. By analyzing the obtained results, the “boundary” (adjacent to high-angle grain boundary) subgrains and the coalescence-induced boundary subgrain clusters were assembled into one group, and the “inside” (non-adjacent to high-angle grain boundary) subgrains into another group (Figure 5). The results given in Figure 7b confirm the previous assumption that coalescence at arbitrary high-angle boundaries develops more intensively compared to the remaining grain volume or at special boundaries (Figure 7c). Thus, the clusters of subgrains with increased dimensions are formed at arbitrary grain boundaries. These clusters serve as energetically favorable nuclei (sites) for further recrystallization. No such effect was observed at a special boundary (Figure 7c). At the end of deformation, the mean subgrain size was 0.375 µm at the incident boundary and 0.270 µm at the special boundary. The evolution of the resulting subgrain cluster at the considered arbitrary grain boundary is demonstrated in Figure 7d for different instants of deformation. In general, the random orientation of the bicrystal with an incident high-angle boundary does not change the nature of the dependencies given in Figure 7; the same is true for the special boundary.

**Figure 8:**
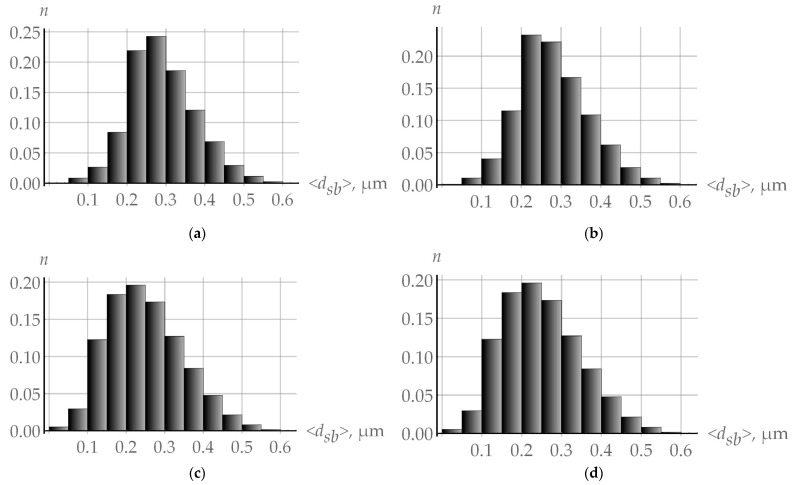
Linear subgrain size distribution histograms plotted based on the results obtained in the framework of the multilevel model with consideration of coalescence at (**a**) high-angle and (**b**) special boundaries and without consideration of coalescence at (**c**) high-angle and (**d**) special boundaries during the quasi-uniaxial deformation test ($\dot{\varepsilon }$ = 10^−5^ s^−1^ and T = 550 K).

Figure 8a shows the size subgrain distribution near the high-angle grain boundary at 10% deformation, which was obtained in the numerical experiment performed at $\dot{\varepsilon }$ = 10^−5^ s^−1^ and T = 550 K. Figure 8b presents the same data for the special boundary $\Sigma_{3}$. Similar histograms are given in Figure 8c,d for the case when coalescence is neglected; the high-angle boundary type has no effect on the results. The analysis of the results revealed that almost the same large subgrain clusters as those inside the grain are formed at the special boundary; that is why they will not be considered below.

**Figure 9:**
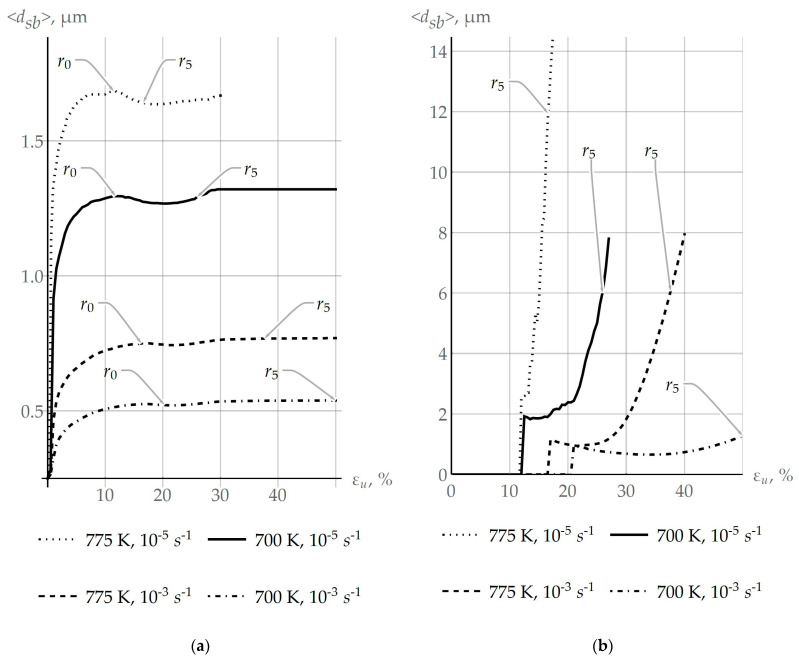
(**a**) Evolution of the mean subgrain size $<d_{sb}>$ and (**b**) recrystallized grains $<d_{gr}>$ in the uniaxial deformation experiment at different deformation velocities and temperatures.

The influence of coalescence on the recrystallization process is the focus of this study, and therefore the results of modeling are given for the deformation velocity $\dot{\varepsilon }$ ranging from 10^−5^ s^−1^ to 10^−3^ s^−1^ and temperatures T from 550 to 775 K. Figure 9a shows how the mean subgrain size changes in the specified ranges of temperatures and deformation velocities. The points corresponding to the onset of recrystallization and the 5% volume fraction of recrystallized material are denoted by symbols “$r_{0}$” and “$r_{5}$”, respectively. In this case, the growth of the mean subgrain size significantly depends on the coalescence process, which is actively implemented at elevated temperatures and low velocities of deformation. It is important now to pay attention to the non-monotonic behavior of the plot displaying the dependence of $<d_{sb}>$ on the deformation intensity. This behavior is explained by the fact that large subgrains are assigned to the category of individual recrystallized grains, provided that criterion (1) is fulfilled. The evolution of the average size of recrystallized grains $<d_{gr}>$ is shown in Figure 9b. The nature of the nonmonotonic curve of the function $<d_{gr}>$ (Figure 9b) is caused by two processes: the transition from subgrains to individual recrystallized grains, and the normal growth of recrystallized grains. Note that the recrystallization criterion (1) is fulfilled first for coarse subgrains. For these subgrains, the effect of the energy of the grain boundary (second term in (1)), at which the recrystallization process slows down, is less pronounced. Thus, a sharp increase in the size of recrystallized grains $<d_{gr}>$ is seen on the graph at the initial instant of recrystallization (Figure 9b). At low deformation velocities of 10^−5^ s^−1^, the growth of recrystallized grains proceeds more intensively than the subgrain transition as a result of fulfilling the recrystallization criterion for small subgrains; the average grain size increases. At 10^−5^ s^−1^, the rate of transition from subgrains to recrystallized grains exceeds the normal grain growth in a certain deformation segment. Based on the results obtained, it can be concluded that the low deformation velocities and elevated temperatures promote both an increase in the size of subgrains at coalescence and the formation of new recrystallized grains.

**Figure 10:**
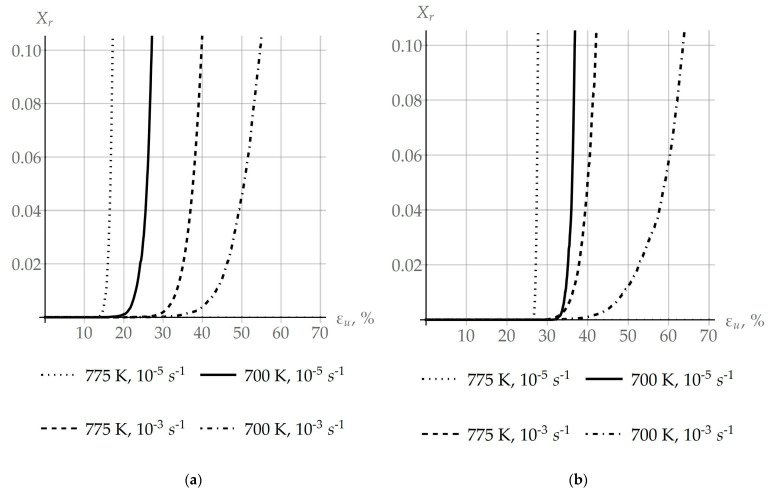
Dependence of the volume fraction of recrystallized material $X_{r}$ on the strain intensity obtained in the experiment on uniaxial deformation at different deformation velocities and temperatures: (**a**) coalescence is considered and (**b**) coalescence is ignored.

The dependence of the volume fraction of recrystallized material $X_{r}$ on the strain intensity of deformations at $\dot{\varepsilon }$ ranging from 10^−5^ to 10^−3^ s^−1^ and at T from 550 to 775 K is given in Figure 10. The value of recrystallized material $X_{r}$ is determined by relations (10)–(12). Figure 10a presents the results of modeling obtained with the consideration of coalescence, and Figure 10b shows these results ignoring coalescence. It can be seen that coalescence induces recrystallization at lower values of deformation and is responsible for the intensive growth of new grains.

This can be attributed to the fact that, due to an increase in the size of subgrains in one of the adjacent grains, coalescence has an impact on the fulfillment of the recrystallization criterion (1), shifting the critical deformation $\varepsilon_{c}$ observed at the onset of recrystallization to lower values.

**Figure 11:**
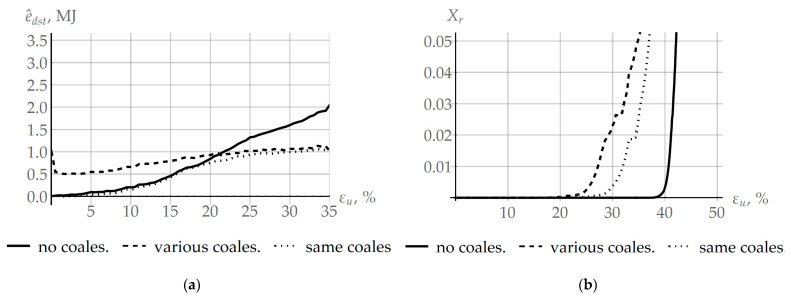
Evolution of the difference in specific stored energy ${\hat{e}}_{dst}$ associated with (**a**) coalescence and (**b**) the volume fraction of the recrystallized material $X_{r}$ on the deformation intensity in the uniaxial deformation experiment.

Depending on the intensity of coalescence in adjacent grains, the coalescence process promotes recrystallization (Figure 3b) or slows it down (Figure 3a). Under the hypotheses accepted in this study, the impact parameters (deformation velocity $\dot{\varepsilon }$ and temperature T) are the same for all considered grains. The coalescence model is determined by two main internal variables: (1) the angle of rotation $\varphi_{c}$, which juxtaposes the crystallographic subgrain lattices occurred at coalescence, and (2) the area s between the adjacent subgrains of the facet. To demonstrate the benefits of the developed model for designing various scenarios about the influence of coalescence on recrystallization, a bicrystal with varying initial subgrain structure was explored. To specify a subgrain structure in the reference configuration, two variants were considered. In the first, a representative volume of subgrains was formed in both grains according to the Rayleigh law. The average subgrain size was 0.25 µm, the mean subgrain misorientation angle was 0.71°, and the axis direction was assumed to be random and uniformly distributed in the sphere. This case is designated in Figure 11 as “same oalescence”; the coalescence process develops in almost the same way in each grain. In the second case, different distributions of subgrain sizes with an average value of 0.2 μm and 0.35 µm and misorientation angles with mean values 0.6° and 1.7° were, respectively, specified (in Figure 11—“various coalescence”) under the Rayleigh law. Note that intensive coalescence was observed in the first grain, which corresponds to the scenario from Figure 3b. Figure 11a shows how the difference in specific energy stored on defects ${\hat{e}}_{dst}$ determined in the computational quasiaxial deformation experiment ($\dot{\varepsilon }$ = 10^−5^ s^−1^, *T* = 700 K) changes in the bicrystal grains under study. The evolution of the volume fraction of recrystallized material is demonstrated in Figure 11b. Since the initial stored energy depends on the density of subgrain boundary dislocations, then, for equal subgrain misorientations, the difference ${\hat{e}}_{dst}$ is practically equal to zero. Additionally, vice versa, in the case of varying initial defect structure, this value was different from zero; its subsequent decrease, shown in Figure 11a, is associated with the coalescence-induced stored energy release. The increase in stored energy, visible on all graphs in Figure 11, is associated with the accumulation of defects inside grains. Despite the fact that coalescence is a process in competition with recrystallization regarding the stored energy, an increase in the level of stored energy eventually leads to the fulfillment of the recrystallization criterion (1). The onset of recrystallization and its subsequent evolution is illustrated in Figure 11b. Recrystallization also causes a decrease in the stored energy, which in turn reduces the rate of accumulation of ${\hat{e}}_{dst}$ (Figure 11a). It can be seen that the implementation of coalescence with equal intensity—“same coalescence”—(the scenario from Figure 3a) slows down recrystallization and increases the critical deformation compared to the coalescence occurred in an inhomogeneous fashion—“various coalescence”—in adjacent grains (scenario from Figure 3b). In both cases, coalescence promotes the earlier onset of recrystallization compared to the case when the coalescence event is ignored. For comparison, the results corresponding to the “no coalescence” scenario are shown in Figure 11a.

## 5. Conclusions

The objective of this study was to develop a multilevel approach to inelastic deformation for modeling the formation of recrystallization nuclei at initial grain boundaries, following the SIBM mechanism and taking into account the effect of coalescence. The previously developed advanced statistical model of inelastic deformation was modified with intent to consider the recrystallization and coalescence processes. Coalescence occurs at the scale level (subgrains level), lower than that of recrystallization (grain level). Coalescence is a competing process with recrystallization for the energy stored on defects, which is the driving force of both processes. As is seen from Figure 11a (the “various coales.” case), a difference in the coalescence intensity on the sides of the grain boundary leads to a decrease of approximately 50% in the storage energy difference at the initial stage of the plastic deformation (about 2.5%). On the other hand, the same coalescence intensity (Figure 11a, “same coales.”) during the deformation up to 20% does not result in a visible deviation of the storage energy difference from the value in the case where coalescence is not considered (Figure 11a, “no coales.”). It is noteworthy that energy release during coalescence occurs in both investigated cases, but the main value determining primary recrystallization is the storage energy, which is shown in Figure 11a. Since coalescence, which is a part of the recovery mechanism, leads to defect structure homogenization and hence storage energy equalization over neighboring grains, one can observe from Figure 11a the storage energy difference reaching a stationary value. Thus, coalescence resulting in the dissociation of subgrain boundaries reduces the density of defects and releases energy. It can be concluded that intensive coalescence on both sides of the grain boundary can be the reason for the difficulty of recrystallization or its complete stop, as evidenced from the computation results presented in Figure 11b. The initial parameters of the subgrain structure (subgrain sizes and misorientation angles) can be chosen to provide a decrease in the storage energy difference without the recrystallization possibility. Such a state of the defect structure and its development scenario are characteristic features of materials with high enough SFE for continuous recrystallization. On the other hand, the realization of coalescence in a low-defect grain, while it is absent in the adjacent, more defective grain, gives rise to more intensive recrystallization at the first stage of this process. Figure 11b shows that, by virtue of coalescence (subgrain coarsening), it is possible to decrease the critical deformation value to initiate recrystallization down to about 20%. Decreases in the critical deformation value for various deformation rates and temperatures can be estimated by results provided in Figure 10. These estimates are 18.08%, 14.65%, 6.05%, and 5.42% for the influence parameters (10^−5^ s^−1^ and 700 K, 10^−5^ s^−1^ and 775 K, 10^−3^ s^−1^ and 775 K, and 10^−3^ s^−1^ and 700 K), respectively. To evaluate the above-described effect of the storage energy release during coalescence, the recrystallization criterion was modified with regard to the SIBM mechanism (Equation (8)). This provides a possibility for evaluating a decrease in the coalescence-induced stored energy. In addition to the energy aspect, coalescence leads to the growth of subgrain sizes. It is shown that coarse subgrain clusters, which further become recrystallization nuclei, are formed at high-angle grain boundaries (Figure 9a). High coalescence intensities at favorable conditions (low deformation rates and high temperatures) contribute to an increase in the average subgrain size from 0.25 µm to about 1.7 µm (Figure 9a). This leads to an increase in the growth rate of recrystallized grains (Figure 9b), which reaches a maximum value of 0.0033 µm/s in the case of deformation with a rate of 10^−5^ s^−1^ and a temperature of 775 K, and minimum 0.00013 µm/s for 10^−3^ s^−1^, 700 K. Unlike arbitrary boundaries with energy of 0.337 J/m^2^, these effects do not appear at special boundaries with reduced energy 0.225 J/m^2^, and thus there is no positive effect of special boundaries on coalescence and the formation of recrystallization nuclei. The results of the multilevel modeling of inelastic deformation in the example of a copper bicrystal demonstrate the capabilities of the developed model for describing the material substructure evolution and the influence of coalescence on the recrystallization process. This model is a component of the multilevel one for describing behaviors of representative volume elements of polycrystalline materials.

## References

[1] Zhao J., Jiang Z. (2018). Thermomechanical processing of advanced high strength steels. Prog. Mater. Sci..

[2] Semiatin S.L. (2020). An Overview of the Thermomechanical Processing of α/β Titanium Alloys: Current Status and Future Research Opportunities. Metall. Mater. Trans. A.

[3] Muramatsu M., Aoyagi Y., Tadano Y., Shizawa K. (2014). Phase-field simulation of static recrystallization considering nucleation from subgrains and nucleus growth with incubation period. Comput. Mater. Sci..

[4] Callister W.D., Rethwisch D.G. (2020). Fundamentals of Materials Science and Engineering: An Integrated Approach.

[5] Nishiyama Z. (1978). Martensitic Transformation.

[6] Gorelik S.S., Dobatkin S.V., Kaputkina L.M. (2005). Recrystallization of Metals and Alloys.

[7] Rollett A., Rohrer G.S., Humphreys J. (2017). Recrystallization and Related Annealing Phenomena.

[8] Panin V.E., Yegorushkin V.E., Makarov P.V., Nemirovich-Danchenko M.M., Demidov V.N., Smolin I.Y., Cherepanov O.I., Psakhie S.G., Negreskul S.I., Zolnikov K.P. (1995). Physical Mesomechanics and Computer Design of Materials.

[9] Panin V.E., Makarov P.V., Nemirovich-Danchenko M.M., Demidov V.N., Smolin I.Y., Cherepanov O.I., Psakhie S.G., Negreskul S.I., Zolnikov K.P., Korostelev S.Y. (1995). Physical Mesomechanics and Computer Design of Materials.

[10] Watanabe T. (2011). Grain boundary engineering: Historical perspective and future prospects. J. Mater. Sci..

[11] Doherty R.D., Hughes D.A., Humphreys F.J., Jonas J.J., Jensen D.J., Kassner M.E., King W.E., McNelley T.R., McQueen H.J., Rollett A.D. (1997). Current issues in recrystallization: A review. Mater. Sci. Eng. A.

[12] Rios P.R., Siciliano F., Sandim H.R.Z., Plaut R.L., Padilha A.F. (2005). Nucleation and growth during recrystallization. Mater. Res..

[13] Huang K., Logé R.E. (2016). A review of dynamic recrystallization phenomena in metallic materials. Mater. Des..

[14] Zhang H.K., Xiao H., Fang X.W., Zhang Q., Logé R.E., Huang K. (2020). A critical assessment of experimental investigation of dynamic recrystallization of metallic materials. Mater. Des..

[15] Zhang J., Yi Y., Huang S., Mao X., He H., Tang J., Guo W., Dong F. (2021). Dynamic recrystallization mechanisms of 2195 aluminum alloy during medium/high temperature compression deformation. Mater. Sci. Eng. A.

[16] Alaneme K.K., Okotete E.A. (2019). Recrystallization mechanisms and microstructure development in emerging metallic materials: A review. J. Sci. Adv. Mater. Devices.

[17] Bailey J.E., Hirsch P.B. (1962). The recrystallization process in some polycrystalline metals. Proc. R. Soc. Lond. Ser. Math. Phys. Sci..

[18] Doherty R.D., Cahn R.W. (1972). Nucleation of new grains in recrystallization of cold-worked metals. J. Common Met..

[19] Vandermeer R.A., Rath B.B. (1989). Modeling recystallization kinetics in a deformed iron single crystal. Metall. Trans. A.

[20] Babu K.A., Prithiv T.S., Gupta A., Mandal S. (2021). Modeling and simulation of dynamic recrystallization in super austenitic stainless steel employing combined cellular automaton, artificial neural network and finite element method. Comput. Mater. Sci..

[21] Derazkola H.A., Garcia E., Murillo-Marrodán A., Fernandez A.C. (2022). Review on modeling and simulation of dynamic recrystallization of martensitic stainless steels during bulk hot deformation. J. Mater. Res. Technol..

[22] Trusov P., Shveykin A. (2019). Multilevel Models of Mono and Polycrystalline Materials: Theory, Algorithms, Application Examples.

[23] Han F., Roters F., Raabe D. (2020). Microstructure-based multiscale modeling of large strain plastic deformation by coupling a full-field crystal plasticity-spectral solver with an implicit finite element solver. Int. J. Plast..

[24] Sun Z.C., Wu H.L., Cao J., Yin Z.K. (2018). Modeling of continuous dynamic recrystallization of Al-Zn-Cu-Mg alloy during hot deformation based on the internal-state-variable (ISV) method. Int. J. Plast..

[25] Zhou G., Li Z., Li D., Peng Y., Zurob H.S., Wu P. (2017). A polycrystal plasticity based discontinuous dynamic recrystallization simulation method and its application to copper. Int. J. Plast..

[26] Zecevic M., Knezevic M., McWilliams B., Lebensohn R.A. (2020). Modeling of the thermo-mechanical response and texture evolution of WE43 Mg alloy in the dynamic recrystallization regime using a viscoplastic self-consistent formulation. Int. J. Plast..

[27] Trusov P., Kondratev N., Podsedertsev A. (2022). Description of Dynamic Recrystallization by Means of An Advanced Statistical Multilevel Model: Grain Structure Evolution Analysis. Crystals.

[28] Hayakawa Y., Szpunar J.A. (1997). A comprehensive model of recrystallization for interstitial free steel. Acta Mater..

[29] Cram D.G., Zurob H.S., Brechet Y.J.M., Hutchinson C.R. (2009). Modelling discontinuous dynamic recrystallization using a physically based model for nucleation. Acta Mater..

[30] Kondratev N.S., Trusov P.V., Podsedertsev A.N. (2021). Multilevel model of polycrystals: Application to assessing the effect of texture and grains misorientation on the critical deformation of the dynamic recrystallization initiation. PNRPU Mech. Bull..

[31] Lebensohn R.A., Tomé C.N., Castaneda P.P. (2022). Self-consistent modelling of the mechanical behaviour of viscoplastic polycrystals incorporating intragranular field fluctuations. Philos. Mag..

[32] Zhao P., Wang Y., Niezgoda S.R. (2018). Microstructural and micromechanical evolution during dynamic recrystallization. Int. J. Plast..

[33] Sarrazola D.A.R., Muñoz D.P., Bernacki M. (2020). A new numerical framework for the full field modeling of dynamic recrystallization in a CPFEM context. Comput. Mater. Sci..

[34] Kondratev N., Trusov P., Podsedertsev A., Baldin M. (2022). Subgrain Coalescence Simulation by Means of an Advanced Statistical Model of Inelastic Deformation. Materials.

[35] Saetre T.O., Ryum N. (1995). On grain and subgrain rotations in two dimensions. Metall. Mater. Trans. A.

[36] Traka K., Sedighiani K., Bos C., Lopez J.G., Angenendt K., Raabe D., Sietsma J. (2021). Topological aspects responsible for recrystallization evolution in an IF-steel sheet—Investigation with cellular-automaton simulations. Comput. Mater. Sci..

[37] Grand V., Flipon B., Gaillac A., Bernacki M. (2022). Simulation of Continuous Dynamic Recrystallization Using a Level-Set Method. Materials.

[38] Choi S.H., Cho J.H. (2005). Primary recrystallization modelling for interstitial free steels. Mater. Sci. Eng. A.

[39] Saetre T.O., Ryum N., Evangelista E. (1991). Simulation of subgrain growth by subgrain rotation: A one-dimensional model. Metall. Trans. A.

[40] Takaki T., Yamanaka A., Tomita Y. (2007). Phase-Field Modeling and Simulation of Nucleation and Growth of Recrystallized Grains. Mater. Sci. Forum.

[41] Li J.C.M. (1962). Possibility of Subgrain Rotation during Recrystallization. J. Appl. Phys..

[42] Rollett A.D. (1997). Overview of modeling and simulation of recrystallization. Prog. Mater. Sci..

[43] Watanabe T. (1984). An approach to grain boundary design for strong and ductile polycrystals. Res. Mech..

[44] Gao S., Hu Z., Duchamp M., Krishnan P.S.R., Tekumalla S., Song X., Seita M. (2020). Recrystallization-based grain boundary engineering of 316L stainless steel produced via selective laser melting. Acta Mater..

[45] Yang X., Wang P., Huang M. (2022). Grain boundary evolution during low-strain grain boundary engineering achieved by strain-induced boundary migration in pure copper. Mater. Sci. Eng. A.

[46] Humphreys J.F. (2004). Nucleation in Recrystallization. Mater. Sci. Forum..

[47] Zhang J.X., Ma M., Liu W.C. (2017). Effect of initial grain size on the recrystallization and recrystallization texture of cold-rolled AA 5182 aluminum alloy. Mater. Sci. Eng. A.

[48] Zhao L.Y., Yan H., Chen R.S., Han E.H. (2021). Orientations of nuclei during static recrystallization in a cold-rolled Mg-Zn-Gd alloy. J. Mater. Sci. Technol..

[49] Doherty R.D. (1974). The Deformed State and Nucleation of Recrystallization. Met. Sci..

[50] Yildirim C., Mavrikakis N., Cook P.K., Rodriguez-Lamas R., Kutsal M., Poulsen H.F., Detlefs C. (2022). 4D microstructural evolution in a heavily deformed ferritic alloy: A new perspective in recrystallisation studies. Scr. Mater..

[51] Nielsen J.P. (1954). Mechanism for the Origin of Recrystallization Nuclei. JOM.

[52] Jones A.R., Ralph B., Hansen N., Cottrell A.H. (1979). Subgrain coalescence and the nucleation of recrystallization at grain boundaries in aluminium. Proc. R. Soc. Lond. Math. Phys. Sci..

[53] Furu T., Ørsund R., Nes E. (1995). Subgrain growth in heavily deformed aluminium—Experimental investigation and modelling treatment. Acta Metall. Mater..

[54] Sandström R. (2022). Formation of Cells and Subgrains and Its Influence on Properties. Metals.

[55] Cahn R.W. (1950). A New Theory of Recrystallization Nuclei. Proc. Phys. Soc. Sect. A.

[56] Dillamore I.L., Smith C.J.E., Watson T.W. (1967). Oriented Nucleation in the Formation of Annealing Textures in Iron. Met. Sci. J..

[57] Sakai T., Belyakov A., Kaibyshev R., Miura H., Jonas J.J. (2014). Dynamic and post-dynamic recrystallization under hot, cold and severe plastic deformation conditions. Prog. Mater. Sci..

[58] Yang Q., Wang X., Li X., Deng Z., Jia Z., Zhang Z., Huang G., Liu Q. (2017). Hot deformation behavior and microstructure of AA2195 alloy under plane strain compression. Mater. Charact..

[59] Wang Y., Zhao G., Xu X., Chen X., Zhang W. (2018). Microstructures and mechanical properties of spray deposited 2195 Al-Cu-Li alloy through thermo-mechanical processing. Mater. Sci. Eng. A.

[60] Wang S., Zhang M., Wu H., Yang B. (2016). Study on the dynamic recrystallization model and mechanism of nuclear grade 316LN austenitic stainless steel. Mater. Charact..

[61] Beck P.A., Sperry P.R. (1950). Strain Induced Grain Boundary Migration in High Purity Aluminum. J. Appl. Phys..

[62] Bailey J.E. (1960). Electron microscope observations on the annealing processes occurring in cold-worked silver. Philos. Mag. J. Theor. Exp. Appl. Phys..

[63] Theyssier M.C., Driver J.H. (1999). Recrystallization nucleation mechanism along boundaries in hot deformed Al bicrystals. Mater. Sci. Eng. A.

[64] Faivre P., Doherty R.D. (1979). Nucleation of recrystallization in compressed aluminium: Studies by electron microscopy and Kikuchi diffraction. J. Mater. Sci..

[65] Hu H. (1962). Direct observations on annealing of a Si-Fe crystal in electron microscope. Trans. Metall. Soc. AIME.

[66] Sandström R. (1977). On recovery of dislocations in subgrains and subgrain coalescence. Acta Metall..

[67] Kuhlmann-Wilsdorf D., Thompson A.W. (1977). Work Hardening in Tension and Fatigue.

[68] Kuhlmann-Wilsdorf D. (1989). Theory of plastic deformation: Properties of low energy dislocation structures. Mater. Sci. Eng. A.

[69] Kuhlmann-Wilsdorf D., Hansen N. (1991). Geometrically necessary, incidental and subgrain boundaries. Scr. Metall. Mater..

[70] Beucia B., Queyreau S., Kahloun C., Chaubet D., Franciosi P., Bacroix B. (2019). Plastic strain-induced grain boundary migration (SIBM) in pure aluminum: SEM in-situ and AFM examinations. Int. J. Plast..

[71] Zhao M., Huang L., Zeng R., Wen D., Su H., Li J. (2019). In-situ observations and modeling of static recrystallization in 300 M steel. Mater. Sci. Eng. A.

[72] Xie B., Zhang B., Ning Y., Fu M.W. (2019). Mechanisms of DRX nucleation with grain boundary bulging and subgrain rotation during the hot working of nickel-based superalloys with columnar grains. J. Alloys Compd..

[73] Jedrychowski M., Bacroix B., Tarasiuk J., Wroński S. (2021). Monte Carlo modelling of recrystallization in alpha Zirconium. Comput. Mater. Sci..

[74] Bellier S.P., Doherty R.D. (1977). The structure of deformed aluminium and its recrystallization—Investigations with transmission Kossel diffraction. Acta Metall..

[75] Sitdikov O., Kaibyshev R. (2001). Dynamic Recrystallization in Pure Magnesium. Mater. Trans..

[76] Zhu S.Q., Ringer S.P. (2018). On the role of twinning and stacking faults on the crystal plasticity and grain refinement in magnesium alloys. Acta Mater..

[77] Bay B. (1970). Subgrain growth on annealing of thin foils of cold-rolled aluminium. J. Mater. Sci..

[78] Doherty R.D., Szpunar J.A. (1984). Kinetics of sub-grain coalescence—A reconsideration of the theory. Acta Metall..

[79] Read W.T., Shockley W. (1950). Dislocation Models of Crystal Grain Boundaries. Phys. Rev..

[80] Ma N., Kazaryan A., Dregia S.A., Wang Y. (2004). Computer simulation of texture evolution during grain growth: Effect of boundary properties and initial microstructure. Acta Mater..

[81] Quey R., Renversade L. (2018). Optimal polyhedral description of 3D polycrystals: Method and application to statistical and synchrotron X-ray diffraction data. Comput. Methods Appl. Mech. Eng..

[82] Kondratev N., Podsedertsev A., Trusov P. (2022). The polycrystals grain structure formation for modified two-level crystal plasticity statistical models. Procedia Struct. Integr..

[83] Kondratev N.S., Trusov P.V. (2018). Modeling of subgrain’s crystallographic misorientation distribution. Nanosci. Technol. Int. J..

[84] Kondratev N.S., Trusov P.V. (2019). Multilevel models of inelastic deformation: Determination of stable low angle boundaries. Mater. Phys. Mech..

[85] Kondratev N.S., Trusov P.V. (2016). Calculation of the intergranular energy in two-level physical models for describing thermomechanical processing of polycrystals with account for discontinuous dynamic recrystallization. Nanosci. Technol. Int. J..

[86] Kronberg M.L., Wilson F.H. (1949). Secondary Recrystallization in Copper. JOM.

[87] Bezverkhy D.S., Kondratev N.S. Application of Coincidence-site Lattice Modification for Grain Boundary Energy Modeling. AIP Conf. Proc..

[88] Bronkhorst C.A., Kalidindi S.R., Anand L. (1992). Polycrystalline plasticity and the evolution of crystallographic texture in FCC metals. Philos. Trans. R. Soc. Lond. Ser. A.

[89] Blaz L., Sakai T., Jonas J.J. (1983). Effect of initial grain size on dynamic recrystallization of copper. Met. Sci..

